# Integrating heterogeneous sequence information for transcriptome-wide microarray design; a Zebrafish example

**DOI:** 10.1186/1756-0500-3-192

**Published:** 2010-07-13

**Authors:** Han Rauwerda, Mark de Jong, Wim C de Leeuw, Herman P Spaink, Timo M Breit

**Affiliations:** 1 Microarray Department & Integrative Bioinformatics Unit, Swammerdam Institute for Life Sciences, Faculty of Science, University of Amsterdam, Amsterdam, The Netherlands; 2 Institute of Biology, Leiden University, Wassenaarseweg 64, 2333 AL, Leiden, The Netherlands; 3 Netherlands Bioinformatics Centre, Nijmegen, The Netherlands

## Abstract

**Background:**

A complete gene-expression microarray should preferably detect all genomic sequences that can be expressed as RNA in an organism, i.e. the transcriptome. However, our knowledge of a transcriptome of any organism still is incomplete and transcriptome information is continuously being updated. Here, we present a strategy to integrate heterogeneous sequence information that can be used as input for an up-to-date microarray design.

**Findings:**

Our algorithm consists of four steps. In the first step transcripts from different resources are grouped into Transcription Clusters (TCs) by looking at the similarity of all transcripts. TCs are groups of transcripts with a similar length. If a transcript is much smaller than a TC to which it is highly similar, it will be annotated as a subsequence of that TC and is used for probe design only if the probe designed for the TC does not query the subsequence. Secondly, all TCs are mapped to a genome assembly and gene information is added to the design. Thirdly TC members are ranked according to their trustworthiness and the most reliable sequence is used for the probe design. The last step is the actual array design. We have used this strategy to build an up-to-date zebrafish microarray.

**Conclusions:**

With our strategy and the software developed, it is possible to use a set of heterogeneous transcript resources for microarray design, reduce the number of candidate target sequences on which the design is based and reduce redundancy. By changing the parameters in the procedure it is possible to control the similarity within the TCs and thus the amount of candidate sequences for the design. The annotation of the microarray is carried out simultaneously with the design.

## Introduction

The best scientific experiments are the ones based on the most recent scientific knowledge. Thus, in expression studies, our detector, i.e. the microarray, preferably would be based on the most recent and complete understanding of the genome and transcriptome. Although the annotation of commercially available microarrays is or can be [1,2] updated on a regular basis, the microarray designs themselves tend to stay unchanged for long periods of time, also due to legacy issues. Furthermore, the microarray design strategy is in many cases proprietary to the microarray manufacturer. Therefore, and apart from the design of the individual probes, for which a variety of software tools exists [3-6], we need a way to translate our knowledge of the genome and transcriptome into a strategy for microarray design for an organism. This is a trivial task neither on the biological nor on a technical level.

The concept of a gene has evolved from a stretch on the genome that encodes one protein to an entity that represents many and complex relations that exist between sequence and biological function. The definition of a gene by Gerstein et al. [7,8] as 'a union of genomic sequences encoding a coherent set of potentially overlapping functional products' allows genes to have an overlapping sequence, to be alternatively spliced and to exert functions other than protein coding. However, it makes a gene less tangible and thus less prone for microarray probe design because in many cases a gene is not just one distinct physical entity.

On a technical level and fuelled by the information from next-generation sequencing experiments, we experience an unremitting flow of new transcription evidence and genome information. This data is used to improve the information in the transcriptome and genome repositories, such as Vega [9] and Ensembl [10] but also can lead to instability in gene assignments such as Unigene. Each repository uses different approaches to define genes and/or transcripts. The differences include the level of confidence that is required for inclusion of an element into a repository, as well as the different algorithms that are used to map transcripts to a genome assembly and to *in silico *predict genes and transcripts [9-13]. Moreover, and depending on the genome and transcriptome at hand, these resources still change considerably from version to version, of which an example is shown in Table 1.

**Table 1:** Number of Transcripts and Genes in Ensembl

Ensembl version	Date	Assembly	# known protein-coding genes	# transcripts
57	March 2010	GRCh37	22,253	142,746

56	Feb. 2009	GRCh37	23,438	140,426

55	July 2009	GRCh37	22,258	101,641

53	March 2009	NCBI36	21,370	62,877

49	March 2008	NCBI36	21,541	48,400

46	Aug. 2007	NCBI36	21,667	44,340

36	March 2006	NCBI36	25,078	21,206

Number of known protein-coding genes and transcripts in the Ensembl human genome releases from March 2006 to March 2010 are tabulated together with the assembly on which these genes and transcripts are mapped.

**Figure 1:**
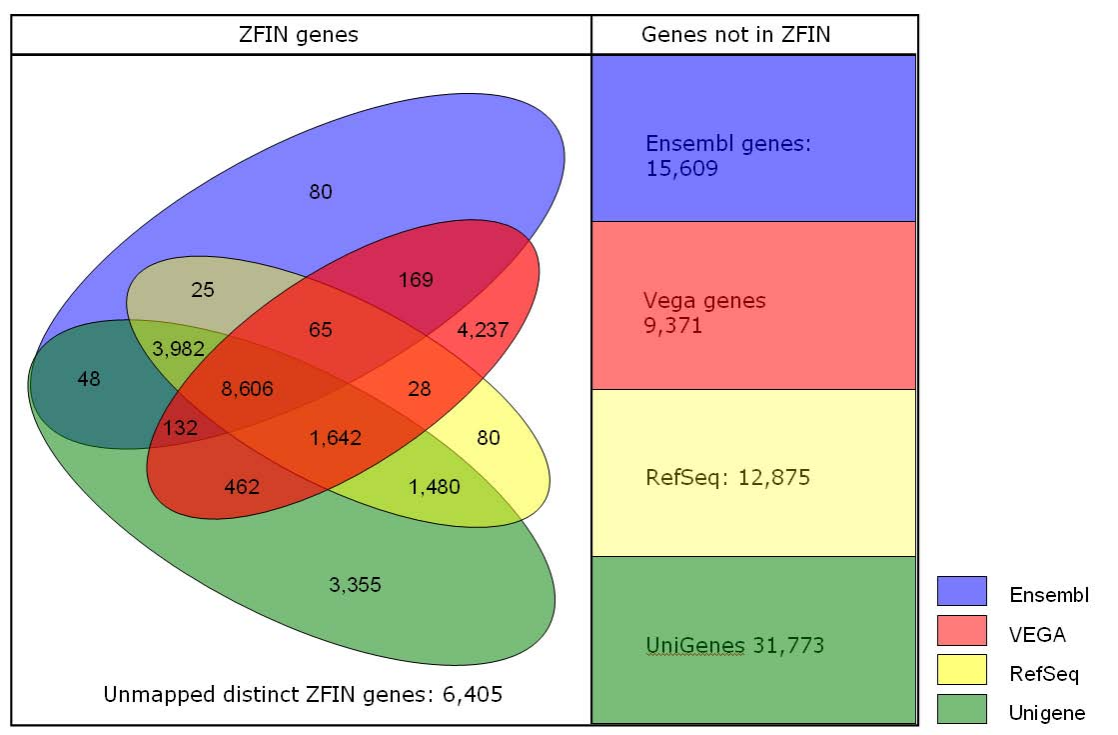
**Mapping of four gene repositories in the Zebrafish Model Organism Database**. Using the mapping tables supplied by the Zebrafish Information Network (ZFIN) [14] (March 2010) on the Unigene, Vega, Ensembl and Refseq genome resources, indicated by colored shades, the number of ZFIN identifiers common to each combination of resources are shown.

Orthogonal to the genome and transcriptome resources are the organism-centric resources, such as the Mouse Genome Informatics (MGI) [14] and the Zebrafish Information Network (ZFIN) http://zfin.org[15], which offer an integrated view on the genome of a selected organism. However, if we were, for instance to base the design of a zebrafish microarray solely on the ZFIN genes, we would exclude a substantial number of genes that is present in one or more of the other resources (Figure 1).

Thus, microarray probes should be designed on transcripts or predicted transcripts, be annotated with gene information and use the most recent transcriptome resources. Because of the exploratory nature of transcriptomics experiments, most scientists wish to detect as many different transcripts as possible, rather than to limit themselves to established transcripts and genes only. The ongoing miniaturization in microarray manufacture also allows such an approach. A simple strategy would be to design probes for all resources separately and put these together on the microarray. However, this approach causes serious difficulties in the expression analysis, such as problems in gene set enrichment and overrepresentation analysis due to redundancy of probes representing the same transcript. Here we will show a strategy to integrate the heterogeneous sequence information for transcriptome-wide microarray design and show the result of our approach for the zebrafish transcriptome.

### Description

**Figure 2:**
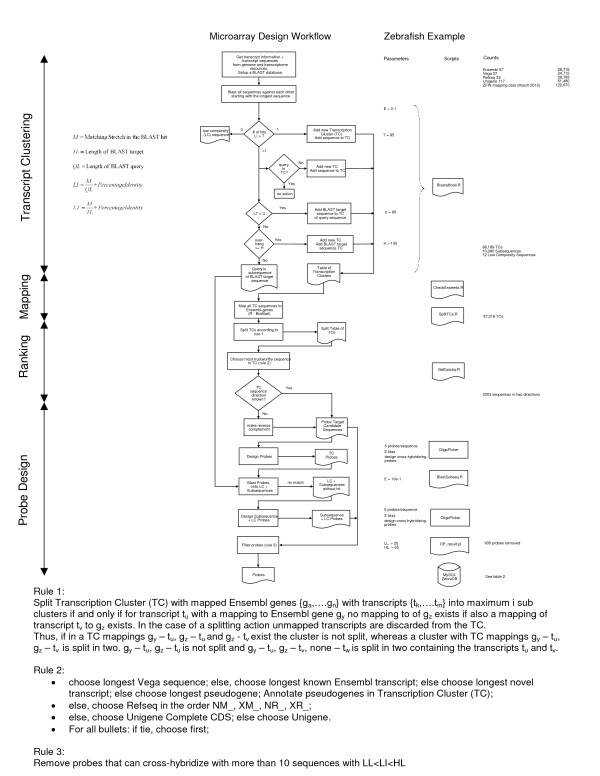
**Microarray Design Workflow**. The diagram of the microarray design workflow is shown together with the rules that are applied at the different stages of the workflow. The parameterization of the Zebrafish array design example, the scripts used, and the counts of the number of entities at each step in the workflow are shown at the right of the figure.

The purpose of the Microarray Design Workflow (Figure 2) is to define, over a set of transcript resources, distinct groups of transcripts that represent distinguishable and non-redundant transcripts. These groups, or Transcription Clusters (TCs), will supply the candidate sequences on which the actual microarray probes are designed. Differences between the TCs should be large enough to make the design of a non-redundant probe likely to be successful. Also, the similarity in a TC should be high enough not to merge biologically different transcripts. The design procedure is organized in 4 steps (Figure 2). First the transcripts are clustered. Secondly the TCs are mapped to a gene assembly and reorganized. In the third step the sequences within cluster are ranked according to trustworthiness. Finally the array can be designed using any oligonucleotide design software.

#### Transcript Clustering

The transcript clustering is started by ordering all sequences by length and, starting with the longest sequence, mapping them onto one another using the BLAST algorithm [16]. A similarity threshold is used as to consider only sequences with a matching part larger than a threshold T (Figure 2). Hence, if a sequence is only similar to itself, a new TC containing that transcript is made. If the sequence can be mapped by using the similarity threshold to more sequences and if those target BLAST sequences that are as long as or longer than the query sequence itself have a matching part larger than a threshold U, the query sequence is added to the TC to which the BLAST target sequence belongs. Query sequences that have a high similarity to the BLAST target sequence but are much smaller may be actual biologically distinct molecules as compared to the BLAST target sequences, e.g. a splice variant or a member of gene family. These sequences are set aside and are further processed in the Array Design step. Thus, if the sequence can be mapped to more sequences, but if those target BLAST sequences that are as long as or longer than the sequence itself have a matching part smaller than a threshold U, the query sequence is categorized as a subsequence unless it contains a non-similar end of at least H nucleotides in comparison with the BLAST target sequence. H is taken sufficiently large as to make the design of a probe possible. This step in the algorithm distinguishes a protruding query sequence from the very similar BLAST target sequence. The parameter H facilitates the distinction between subsequences for which probes might be designed (see the Array Design paragraph) and protruding sequences that are organized by introducing a new TC for which a probe must be designed. If the nucleotide order and composition of a sequence has low complexity, no BLAST hits are returned. These sequences are marked as low-complexity (LC) sequences and are discarded from the design.

#### Transcript Mapping

In order to make a gene annotation for each TCs, the table of TCs is mapped to Ensembl using R-BioMart [17]:. The TC is split if its sequences map to more than one Ensembl gene (Figure 2, Rule 1). In that case, sequences without a mapping to Ensembl are discarded. If a sequence is mapped to more than one Ensembl gene, the TC is only split, if this does not introduce redundancy, i.e. different TCs containing identical transcripts.

#### Transcript Ranking

Next the most-trustworthy sequence in a TC is chosen for the actual probe design. Resources that apply a higher level of biological evidence are deemed to have a higher trustworthiness and sequences in a resource that are annotated based on biological evidence are chosen over transcripts that are *in silico *predictions (Figure 2, Rule 2). For instance: Ensembl transcripts are prioritized in the order 'known', 'novel' and 'pseudogene'. RefSeq sequences are chosen in the order of their prefix NM_ (mature messenger RNA transcripts), XM_ (model mRNA), NR_ (non-coding transcripts) and XR_ (model non-coding transcripts). If there are more UniGenes in the TC, the 'complete cdss' are favored. In all cases, if there is a draw, the longest sequence is taken and if then still no decision can be made the first sequence is chosen. If the 5' to 3' direction of EST-based UniGenes is not known, also the reverse complement of the candidate sequence is made. For a different organism or for a different choice of transcript resources this procedure can be easily adapted.

#### Array Design

With the resulting list of probe target candidate sequences, microarray probes are designed. Next, the designed probes are mapped onto the subsequences using the BLAST algorithm. Subsequences that do not show any similarity with a probe are subjected to a second probe design run.

The final step of this strategy is only applicable to procedures in which the probe design software also can produce cross-hybridizing probes. Probes representing different TCs that cross-hybridize with a high identity can be grouped together, i.e. these probes can be attributed to a specific sequence that is common to a group of TCs. However, probes with a large number of low to medium cross-hybridization events are not interpretable and can be removed from the design (Figure 2, Rule 3).

All scripts used in this procedure are written in R or Perl and are available via our website http://staff.science.uva.nl/~rauwerda/resource_integration_array_design. The script, in which the TCs are constructed, uses a local installation of BLAST and is computationally the most expensive step in the procedure. The computational requirements for the actual probe-design depend on the software used and whether the design of cross-hybridizing probes is included.

### A Zebrafish Example

**Figure 3:**
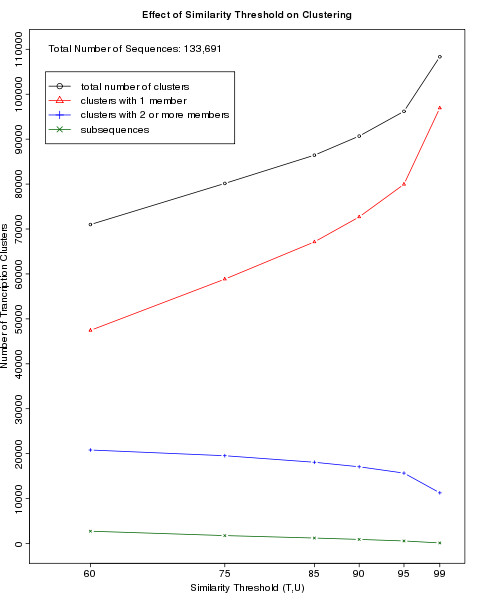
**Effect of the Similarity Threshold on Transcript Clustering**. The result of the mapping of the sequences of Ensembl 57, Vega 37, RefSeq 39, and UniGene 117 onto each other using the procedure as specified in the Description section and Figure 2 is shown. Similarity thresholds T and U were varied from 60 to 99 but were kept equal to one another in each mapping run. In total 133,691 sequences have been mapped. Depicted are the total number of clusters, the clusters with 1 and the clusters with 2 or more members and the number of subsequences. At T = U = 60 45% of all TC sequences is clustered into clusters with 2 or more members. This percentage drops to 12% at T = U = 99. This decrease is due to the higher stringency with respect to the identity of the BLAST query to the BLAST target sequences (T) as well as to the higher limit at which a smaller BLAST query sequence is added to the TC of the larger BLAST target sequence (a higher U facilitates the calling of subsequences). At T = U = 95 a sharp rise in the number of clusters with only one member is observed. However, the increase of the single member clusters is much larger than the increase of the number of subsequences.

The sequence repositories chosen for our Zebrafish Microarray Design: Ensembl 57, Vega 37, RefSeq 39, and UniGene 117, contain 133,691 sequences. Also genome information of ZFIN has been used. In order to establish the behavior of the algorithm on this data we have carried out a parameter-sweep experiment of six mappings using similarity thresholds ranging from 60 to 99 (Figure 3). The total number of clusters produced by the algorithm increases linearly until a similarity threshold T = U = 85 and starts to increase at a higher rate at higher values of T. A sharp decrease in number of clusters with more than 2 members or more is observed a T = U = 95. Additional file 1 lists the tabulated results of these mappings.

For this array design we have chosen to include cross-hybridizing probes. Therefore we have chosen to be rather strict on the similarity threshold, but avoid a high increase rate of the number of clusters. Hence similarity parameters T and U and the overhang parameter H were set as 95, 95 and 100 respectively. The first BLAST procedure produced 96,189 TCs, 10,080 sub-sequences and 12 low-complexity sequences were produced. The splitting of TCs according to the Ensembl gene classification resulted in 97,316 TCs. 2,303 sequences for which the 5'-3'-direction could not be established were designed in both possible transcript directions. Afters the second BLAST procedure, in which the TC probes were aligned against the subsequences (Figure 2), 5,299 subsequences remained to be designed.

Oligopicker [4] has been used to design up to 5 probes per sequence with a 3' sequence preference. A probe is considered to cross-hybridize, if it contains a stretch of 16 nucleotides or has a bitscore higher than 32.5. If only cross-hybridizing probes could be designed, such a probe was added to the design, together with the information to which other sequences this probe can cross-hybridize.

The construction of the TCs took less than a day on a 2.8 GHz Dual-Core AMD Opteron 2220 machine. The microarray design has been carried out on a 20 node Pentium-D computer cluster and took 10 hours.

The results of the zebrafish microarray probe design are summarized in Table 2. For 43% (41,477) of the TC sequences we were able to design 1-5 unique probes, for 56% (54,461) only cross-hybridizing probes could be designed, and for approximately 1.5% no probe could be designed. 935 probes have been removed from the design, because of their potential to massively cross-hybridize with a low to medium stringency.

**Table 2:** Characteristics of the Zebrafish Microarray Design

	TC uni-directional	TC 2 bi-directional	Sub-sequences	Vega 37	Ensembl 57	RefSeq 39	UniGene 117
Probe target candidate sequences	97,316	2,303	5,299	*22,412*	*20,728*	*20,533*	*41,245*

TCs/sequences with 5 probes	3,397	67	0	*821*	*503*	*935*	*1,205*

TCs/sequences with 4 probes	2,213	139	0	*328*	*199*	*394*	*1,431*

TCs/sequences with 3 probes	4,015	233	0	*714*	*309*	*618*	*2,607*

TCs/sequences with 2 probes	8,080	389	1	*1,558*	*676*	*1,138*	*5,098*

TCs/sequences with 1 probe	23,772	947	23	*5,446*	*2,246*	*3,049*	*14,001*

**TCs/sequences without cross-hybridizing probes**	**41,477**	**1,775**	**24**	*8,867*	*3,933*	*6,134*	*24,342*

TCs/sequences with a cross-hybridizing probe	54,461	484	4,620	*13,209*	*16,330*	*13,954*	*16,072*

**Total queried TCs/sequences**	**95,938**	**2,259**	**4,644**	*22,076*	*20,263*	*20,088*	*40,414*

The rows represent the number of candidate sequences and the design result of OligoPicker [4]. The columns represent the number of TCs in one direction, the number of TCs presented to the oligo-design software as reverse complement, and the number of subsequences. The last four columns show the origin of the sequences on which the probe design has been based.

**Figure 4:**
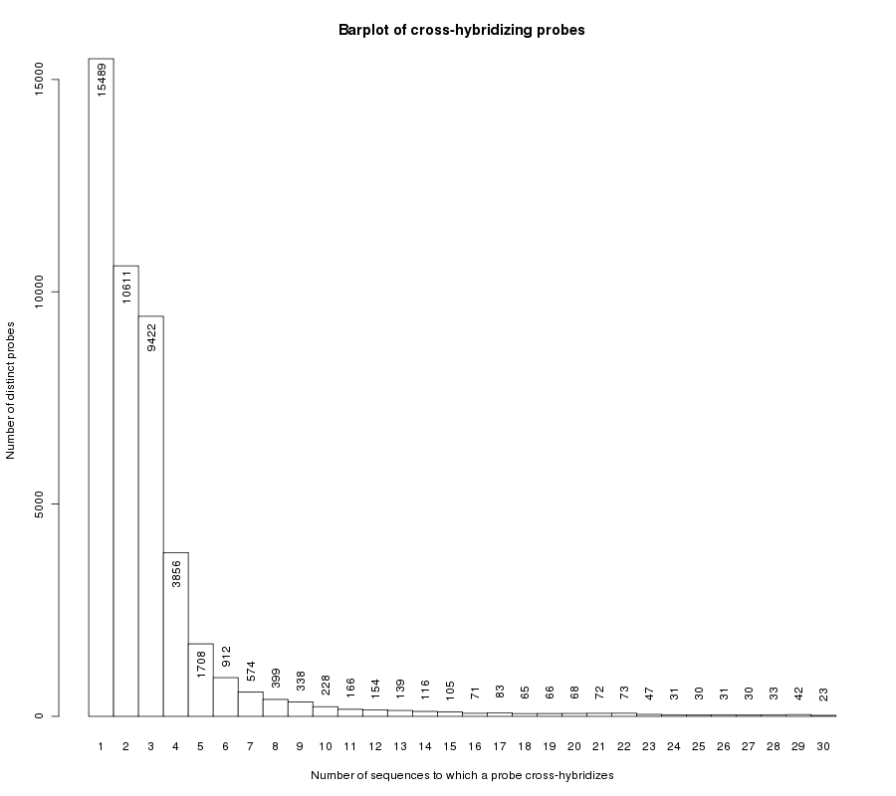
**Barplot of cross hybridizing probes**. Barplot of TC-based uni-directional cross-hybridizing probes. Shown are the probes that cross-hybridize to 30 sequences or less; in or on top of the histogram bars the number of probes is displayed.

We have organized the design information in several additional files: Additional file 2, all non cross-hybridizing probes are tabulated together with their sequences, the characteristics of the transcript the probe is designed on, the other sequences in the TC and the Ensembl genes and Ensembl transcripts mapped onto this TC; Additional file 3, all TCs are given that are queried by non cross-hybridizing probes along with the identifiers of the transcripts and the probe(s); Additional file 4, all cross-hybridizing probes are tabulated together with their sequences, the characteristics of the transcript the probe is designed on, the other sequences in the TC, the Ensembl genes and Ensembl transcripts mapped onto this TC and the TCs to which they cross-hybridize; For a number of TC-pairs no probe could be designed that distinguishes between the members of the pair. In Additional file 5, 10757 probes are tabulated that query two or more of such TCs or subsequences. To indicate the extent of cross hybridization we summarized the number of sequences to which probes cross-hybridize in Figure 4. 38% (15,296) uni-directional cross-hybridizing TC-based probes cross hybridize just to one sequence. 55% (29,797) of all cross-hybridizing probes have only perfect hits to the sequences they cross-hybridize with (Additional file 6). In total, we have designed 126,632 probes in this whole-transcriptome Zebrafish Microarray Design.

### Concluding Remarks

The workflow presented here facilitates the integration of heterogeneous sequence information for transcriptome-wide microarray design and minimizes by construction of Transcription Clusters, the redundancy of transcripts represented on the microarray by probes. Together with the microarray design, the annotation of the microarray is drawn up. Inherently to biology, some probes can never be mapped to individual genes. However, with this approach, all information which transcripts and genes a probe refers to is available. In this zebrafish example we have chosen to also design cross-hybridizing probes. If the research question that has to be answered by the microarray experiment does not need to investigate the biological mechanism at hand, such as in biomarker studies, these cross-hybridizing probes can prove to be quite useful.

With the presented workflow we have developed a tool for microarray design that allows the use of as many heterogeneous genome resources as desired. The easy to design up-to-date microarrays in the current era of high-density custom-designed microarrays makes this workflow a valuable tool for whole-transcriptome studies.

## Competing interests

The authors declare that they have no competing interests.

## Authors' contributions

HR specified and implemented the algorithm; WCdL, MdJ, HPS and TMB all participated in the specification of the strategy.

## Supplementary Material

Additional file 1 **Effect of the Similarity Threshold on Transcript Clustering**. Tabulated are the total number of clusters, clusters with 1, 2, 3, 4 and more than 4 members and the number of subsequences that result from a clustering with a similarity threshold T = U of 60, 75, 85, 90, 95 and 99 respectively. Click here for file

Additional file 2 **Zebrafish Microarray Design - Non Cross-hybridizing Probes**. All non cross-hybridizing probes are tabulated together with their sequences, the TC-id, the transcript the probe is designed on, whether the probe is designed on the sequence given by the sequence resource or is designed on the reverse complement, start of the probe on the transcript, information from the sequence resource, gene symbol, description, chromosome, strand, genomic position, other TC members, Ensembl Gene, and Ensembl Transcript. Click here for file

Additional file 3 **Zebrafish Microarray Design - TCs queried by Non Cross-hybridizing Probes**. all TCs are given that are queried by non cross-hybridizing probes. Tabulated are TC ids, probe ids, members of the TC and the Ensembl gene(s) and transcript(s) to which the TC is mapped. Click here for file

Additional file 4 **Zebrafish Microarray Design - Cross-hybridizing Probes**. all cross-hybridizing probes are tabulated together with their sequences, the TC-id, the transcript the probe is designed on, whether the probe is designed on the sequence given by the sequence resource or is designed on the reverse complement, start of the probe on the transcript, information from the sequence resource, gene symbol, description, chromosome, strand, genomic position, other TC members, Ensembl Gene, Ensembl Transcripts and between brackets: cross-hybridizing potential to transcript, TC with the cross-hybridization bitscore. Subsequences are abbreviated by 'subs'. Click here for file

Additional file 5 **Zebrafish Microarray Design - Probes designed on more than one TC**. Tabulated are the probes that only could be designed to more than one TC. In the second column the TCs are given between brackets, together with the TC sequence on which the probe has been designed. Subsequences are abbreviated by 'subs'. Click here for file

Additional file 6 **Zebrafish Microarray Design - Perfect hit only cross-hybridizing probes**. Tabulated are the probes that have an exclusive 100% similarity to the probes they cross-hybridize with. The probes are given together with the sequences they are designed on and between brackets the TCs they cross-hybridize with plus the sequence the TC has been designed on. Subsequences are abbreviated by 'subs'. Click here for file
